# Gender-Specific Impact of Cadmium Exposure on Bone Metabolism in Older People Living in a Cadmium-Polluted Area in Thailand

**DOI:** 10.3390/ijerph14040401

**Published:** 2017-04-10

**Authors:** Muneko Nishijo, Kowit Nambunmee, Dhitiwass Suvagandha, Witaya Swaddiwudhipong, Werawan Ruangyuttikarn, Yoshikazu Nishino

**Affiliations:** 1 Department of Public Health, Kanazawa Medical University Hospital, Uchinada 920-0293, Japan; ynishino@kanazawa-med.ac.jp; 2 Health Evaluation Center, Kanazawa Medical University, Uchinada 920-0293, Japan; 3 School of Health Science, Mae Fah Luang University, Chiang Rai 57100, Thailand; kowit.nam@mfu.ac.th; 4 Environmental Science Program, Faculty of Science, Chiang Mai University, Chiang Mai 50200, Thailand; dhitiwass@gmail.com; 5 Mae Sot General Hospital, Mae Sot District, Tak Province 63110, Thailand; swaddi@hotmail.com; 6 Department of Forensic Medicine, Faculty of Medicine, Chiang Mai University, Chiang Mai 50200, Thailand; ruangyuttikarn@gmail.com

**Keywords:** blood cadmium, urinary cadmium, bone resorption marker, bone formation marker, whole parathyroid hormone, vitamin D binding protein, Thailand

## Abstract

To elucidate the influence of cadmium exposure on bone metabolism, associations between urinary/blood cadmium and bone resorption/formation markers were investigated in older cadmium exposed men and women. Increased urinary cross-linked N-telopeptide of type I collagen (NTx), a bone resorption marker, was found to be associated with increased levels of parathyroid hormone, fractional excretion of calcium, and urinary/blood cadmium after adjusting for confounding factors in men. In women, urinary NTx was significantly associated with only urinary cadmium and a strong relationship with increased fractional excretion of calcium. Risk for bone metabolic disorders, indicated by high urinary NTx, significantly increased in men with blood cadmium ≥ 10 μg/L or urinary cadmium ≥ 10 μg/g creatinine. Increased osteocalcin level was significantly associated with increased blood cadmium in men. In conclusion, cadmium exposure appeared to have an influence on bone remodeling both bone resorption and formation in this population of older Thai men, and blood cadmium was more closely associated with bone metabolism than urinary cadmium.

## 1. Introduction

Cadmium (Cd) is a toxic heavy metal and is a known environmental pollutant causing chronic adverse health effects on the kidney and bone of residents in Cd-polluted areas [1]. Itai-itai disease (Ouch-ouch disease) is the severest form of Cd poisoning reported in the Jinzu River basin, Toyama, Japan, and is characterized clinically by osteomalacia with severe bone pain caused by advanced renal tubular dysfunction from extremely high exposure to cadmium [2]. In Japan, Kido et al. reported decreased bone mineral density (BMD) compared with controls and associations with renal tubular dysfunction indicated by urinary β2-microglobulin in men and women aged ≥ 50 years old in the Cd-polluted Kakehashi River basin [3]. A Swedish research group conducted the OSteoporosis-CAdmium as a Risk factor (OSCAR) study on men occupationally exposed to Cd and reported a significant association between blood Cd and BMD [4]. Nawrot et al. [5] reported that osteoporosis risk was associated with increased urinary Cd and increased urinary calcium (Ca) excretion in middle-aged male Belgian workers, but that renal tubular dysfunction was not always found in men with low BMD, suggesting direct toxic effects of Cd exposure on bone metabolism in men. They also reported that increased urinary Cd was associated with increasing urinary bone resorption markers in women without renal tubular dysfunction, indicating a direct osteotoxic effect of Cd with increased Ca and alterations in serum parathyroid hormone (PTH) and calcitonin.

Environmental contamination with Cd has been reported in Mae Sot District, Tak Province located in Northwestern Thailand and rice paddy fields in the 12 villages of Mae Sot District were found to contain markedly elevated levels of Cd [6]. Prolonged consumption of rice contaminated with Cd is believed to be a cause of excessive Cd burden on the body, demonstrated by significantly increased urinary Cd in Mae Sot inhabitants [7]. Mae Sot people who live in the contaminated area have reported renal tubular dysfunction indicated by α1-, β2-microglobulin (MG), and N-acetyl-β-D-glucosamidinase (NAG) associated with urinary Cd [8]. Swaddiwudhipong et al. [9] reported that increased serum creatinine (Crea) and decreased glomerular filtration rate was associated with increased urinary Cd in inhabitants in the same contaminated area in Mae Sot with urinary Cd ≥ 5 μg/g Crea. In our own previous research, we found an imbalance in Ca reabsorption because of remarkable renal tubular dysfunction that leads to increased bone resorption markers in residents in Mae Sot aged ≥50 years old, particularly female residents including premenopausal women [10].

The relationship between Cd exposure and bone markers mediated by PTH, which controls Ca metabolism, has not been investigated in Mae Sot residents. Additionally, vitamin D binding protein (VitDBP), which is a carrier protein of Vitamin D and is closely associated with renal functions, was not examined in our previous report. In the current study, the influence of Cd exposure on bone remodeling was evaluated in older residents including only postmenopausal women using biochemical markers of bone and Ca metabolism including whole PTH and urinary VitDBP.

## 2. Materials and Methods

### 2.1. Study Area

The study area was a Cd-contaminated area in Mae Sot District, Tak Province, Thailand, with large paddy fields receiving irrigated water from two creeks named Mae Tao and Mae Ku. Both creeks pass through a zinc rich area where a zinc mine had been actively operating for more than 20 years. Cd concentrations were low in the samples collected from the creeks before reaching the zinc area. Concentrations increased greatly when passing through the zinc mine area, and then reduced according to distance. Cd-contaminated areas were estimated to be about 13,200 rais (approx. 1600 m^2^) of paddy fields affecting 12 villages with a total population of 12,075 in 2004. Eighty-five percent of 1090 paddy soil samples had Cd levels higher than the level recommended by the European Union (3 mg Cd/kg) [11], and more than 90% of rice grain samples contained Cd, exceeding the maximum permissible level for rice grain (0.2 mg Cd/kg) determined by the Codex Committee on Food Additives and Contaminants [6]. The level was also above that considered safe for a staple food (0.1 mg/kg) [12].

### 2.2. Study Participants

In total, there was 705 participants whose urinary Cd concentrations was >5 µg/g Crea identified in Cd exposure surveys in 2004 and 2005 [7] and those participating in a health impact survey in 2007 conducted in a Cd-polluted area in Mae Sot, Thailand [13,14]. The population of the current study was 130 inhabitants (61 men and 69 women) who were aged ≥ 65 years and who were taken from participants in the 2007 health impact survey whose serum volume was enough for the entire PTH measurement. Female participants were all postmenopausal women more than 10 years after the last menstruation and none had hormone replacement therapy (HRT) nor treatment of osteoporosis using active vitamin D metabolite or its analog. Blood and urine of the subjects were analyzed levels of urinary and blood Cd, urinary VitDBP, and bone-related biomarkers, including serum whole PTH.

Informed consent was obtained from all participants before surveys. The study protocol was conducted in accordance with the Declaration of Helsinki and approved by the Institutional Review Board and Ethical Committee of the Kanazawa Medical University, Japan (License No. E18).

### 2.3. Sample Collection and Measurement of Biomarkers

The morning urine specimen was collected in polyethylene bottles after participants underwent physical examination and anthropometric measurements. Venous blood was drawn from participants in the fasted state, and serum was obtained as soon as possible by centrifugation. Urine and blood samples were divided into several aliquots (3–5 mL each), with some being stored at −20 °C until measurement of biomarkers. Urinary Ca, phosphorus (P), and creatinine (Crea) were determined using colorimetric assays (O-Cresolphthalein method for Ca, phosphomolybdic acid method for P, and enzyme assay for Crea; Clinimate test kit; Sekisui Medical, Tokyo, Japan) using an automated analyzer (BioMajesty JCA-BM1650, JEOL, Tokyo, Japan). Ca, P, and Crea in serum were measured using an auto analyzer at Mae Sot Hospital. Serum osteocalcin (OC), which is a bone formation marker, and serum whole PTH was measured by immunoradiometric assay. Urinary cross-linked N-telopeptide of type I collagen (NTx), which is a bone resorption marker, was measured by an enzyme-immunoassay and expressed as nM of bone collagen equivalent per mM of urinary Crea. VitDBP in urine was measured by ELISA (Quantikine human vitamin D binding protein ELISA Kit, R&D Systems, Minneapolis, MN, USA).

### 2.4. Cd Measurement in Urine and Blood

Urinary Cd was measured to determine the Cd body burden using a flameless atomic absorption spectrophotometer (Model AAS-6300, Shimadzu, Kyoto, Japan). Palladium chloride (20 ppm) in 5% nitric acid solution was used as a modifier for urinary Cd quantification. For quality control and validation of the analytical method, urine standard reference material No.2670 (The National Institute of Standards, Washington, DC, USA) was used, which had the obtained geometric mean ± geometrical standard deviation of urinary Cd at a concentration (n = 15) of 5.05 ± 0.22 µg/g Crea, and the certified value was 4.86 ± 0.08 µg/g Crea. The urinary Cd concentration was corrected to the urinary Crea concentration measured using an enzyme assay (Cica liquid–S; Kanto Chemical Co., Reagent Division, Tokyo, Japan). The levels of Cd > 5 µg/g Crea was considered the cutoff level for positive high Cd exposure.

Cd in whole blood was also measured with an atomic absorption spectrophotometer using supernatant after mixing with 5% nitric acid solution and centrifugation [15]. Determination of the control blood (Lot No. 620302, Behring Institute, Dresden, Germany) was observed to be in the confidence range of assigned value. The detection limit of our method was 0.2 μg/L, which was the suspected 0 value for Cd on the calibration curve.

### 2.5. Statistical Analysis

SPSS software (Ver. 21, IBM Inc., New York, NY, USA) was used for statistical analyses. All determined variables, except for age, body size, and whole PTH, were logarithmically transformed because of their approximately log-normal distributions before statistical analysis, then converted back to the normal scale for demonstration. Mean comparisons of age and biomarkers between men and women were tested using the Student’s t-test. A non-parametric analysis method (Spearman’s rho) was used for simple correlations between bone markers and exposure and renal markers. Multiple linear regression analysis was performed to analyze relationships between bone metabolic markers and urinary or blood Cd levels after adjusting for age, body mass index (BMI), current smoker (1: yes, 0: no), current drinker (1: yes, 0: no), serum whole PTH, serum Crea, fractional excretion of calcium (FECa), and urinary VitDBP. To investigate adjusted risk (odds ratio) for bone metabolic disorders from high Cd exposure, a logistic regression model was used with the same covariates as regression analysis. A *p*-value <0.05 was considered statistically significant.

## 3. Results

### 3.1. Bone Metabolism Factors and Cd Exposure

#### 3.1.1. Comparison of Bone Metabolism Factors and Cd Exposure between Men and Women

Means with SD for age, body size, bone markers, whole PTH, serum and urinary Crea, urinary VitDBP, renal excretion markers of Ca and P, and Cd exposure markers were compared between men and women (Table 1). Urinary NTx is a biomarker to estimate the risk of bone metabolic disorders with cut-off values of 66.2 for men and 89.0 for postmenopausal women in general population [16]. Prevalence of bone metabolic disorders defined with these cut-off values of urinary NTx in men and women is shown in Table 1. The prevalence of persons with elevated serum OC with a cut-off value of 12.7, which is in the upper range of healthy persons, is also shown. Urinary NTx was significantly higher, and fractional excretion of phosphorus (FEP) and serum Crea were significantly lower in women compared with men. Although there was no difference in urinary Cd levels between genders, blood Cd in men was significantly higher than that in women.

#### 3.1.2. Correlation between Bone Markers and Markers of Cd Exposure and Ca Metabolism

Simple correlation coefficients (Spearman’s rho) of bone markers with markers of Cd exposure and Ca metabolism are shown in Table 2. In men, FECa significantly correlated with urinary NTx and whole PTH, and serum Crea significantly correlated with serum OC. In women, urinary VitDBP, FECa, urinary Cd, and blood Cd significantly correlated with urinary NTx, although whole PTH and serum Crea inversely correlated with urinary NTx. Serum Crea and urinary VitDBP correlated with serum OC in women. The results suggested that Ca metabolism-related factors including whole PTH and renal markers including VitDBP were associated with increased bone markers.

### 3.2. Adjusted Associations between Bone Markers and Cd Exposure

#### 3.2.1. Associations between Urinary NTx and Urinary and Blood Cd

To clarify the effects of the urinary Cd or blood Cd on urinary NTx after controlling covariates (age, BMI smoking habit, whole PTH, serum Crea, FECa, and urinary VitDBP), multiple regression analyses were performed in men and women (Table 3). In men, urinary Cd showed a significantly positive association with urinary NTx after controlling covariates including smoking, whole PTH, and FECa, which were significantly associated with urinary NTx. Blood Cd was significantly associated with urinary NTx after controlling covariates in men. In women, urinary Cd significantly correlated with urinary NTx (β = 0.243, *p* = 0.026), but the association between urinary NTx and FECa was stronger (β = 0.420, *p* < 0.001) than that with urinary Cd. Blood Cd did not significantly correlate with urinary NTx, although FECa correlated with urinary NTx in women.

#### 3.2.2. Associations between Serum OC and Urinary and Blood Cd

Multiple regression analyses of serum OC with Cd exposure markers and covariates were also performed in both sexes (Table 4). In men, only blood Cd, not urinary Cd, was found to be significantly associated with serum OC after controlling covariates including whole PTH and serum Crea, which significantly correlated with serum OC in Table 2. In women, neither urinary Cd nor blood Cd was associated with serum OC and serum OC and urinary VitDBP was not significantly associated with OC.

#### 3.2.3. Risk (Odds Ratios) for Bone Metabolic Disorders Indicated by High Urinary NTx, Ca Metabolism, and Cd Exposure Factors

Odds ratios for the risk for bone metabolic disorders from factors related to Ca metabolism and Cd exposure were analyzed using a logistic regression model with covariates including age, smoking and drinking habits, and BMI (Table 5). When urinary NTx ≥ 66.2 nM was defined as osteopenia bone metabolic disorders, in men, the odds ratio for the risk for bone metabolic disorders from blood Cd significantly increased as well as FECa and urinary VitDBP, but urinary Cd was not significantly associated with bone metabolic disorders. In women, only the odds ratio for the risk for bone metabolic disorders from FECa significantly increased, and neither Cd exposure indices were associated with bone metabolic disorders. When bone metabolic disorders was defined as urinary NTx ≥ 89.0 nM, the odds ratio for bone metabolic disorders from blood Cd was significantly increased as well as FECa in men, but the increased odd ratio from urinary Cd was not significant. In women, there was no factor significantly associated with bone metabolic disorders.

## 4. Discussion

In older men, increased urinary NTx, a bone resorption marker, was associated with increased PTH, FECa, and urinary and blood Cd after adjusting for confounding factors. In older (postomenopausal) women, urinary NTx was significantly associated with increased FECa and urinary Cd, with the stronger relationship observed with increased FECa. Increased OC was significantly associated with increased blood Cd in men, but it was not associated with any Cd exposure markers in women. Risk for bone metabolic disorders, indicated by high urinary NTx, was significantly increased with Cd exposure markers, particularly with blood Cd ≥10 μg/g L in men, suggesting that older men with high Cd exposure are at increased risk for bone metabolic disorders in this Thai population.

Urinary Cd is often used as a chronic exposure marker and is reported to correlate well with Cd in the kidney. Blood Cd can reflect recent Cd exposure that has transferred from the liver to other organs including the kidney and is often seen in factory workers exposed to Cd at very high concentrations [1]. In the current study, blood Cd levels were significantly higher in men compared with women, with the rate of blood Cd ≥ 10 μg/L being 42.6% in men and 7.2% in women. This suggested that men had current and previous exposure to high level of Cd. There were no differences in smoking rates and numbers of years as a resident in the study area between men and women. This suggested that men had current and previous exposure to high level of Cd. There were no differences in smoking rates and numbers of years as a resident in the study area between men and women. In men, blood Cd was associated with increased risk for osteopenia and with increased bone resorption markers, more so than urinary Cd, suggesting that blood Cd might directly influence collagen metabolism of bone to increase the release of NTx into blood and urine.

Consistent with the findings of the current study, Jarup et al. [4] reported that decreased BMD was associated with blood Cd exposure levels in Swedish factory workers, 95% of whom were men, suggesting that men had a risk for osteoporosis as well as women, and blood Cd showed a greater association with BMD compared with urinary Cd. It was reported that, in the middle-aged male workers in Belgium, the risk for osteoporosis, indicated by decreased BMD, increased in a dose-dependent manner; however, in that study, the exposure marker associated with BMD was urinary Cd [5]. In the same target population in Thailand as the current study, Limpatanachote et al. [17] reported that lower BMD was associated with increased urinary Cd in women with urinary Cd ≥5 μg/g Crea. Inconsistent results in men in that compared with our own BMD study might be related to the higher sensitivity of the bone resorption marker compared with BMD measurement using ultrasound of the calcaneus bone.

In the current study, FECa correlated well with urinary NTx, particularly in women, and significant positive associations between urinary NTx and urinary Cd were observed after controlling FECa in women as well as in men. However, blood Cd was not associated with urinary NTx, with a stronger association with FECa being found in women. Increased urinary excretion of Ca was reported to be associated with increased Cd in the kidney in women [18], suggesting Cd exposure may influence bone metabolism mainly through increased Ca excretion into the urine in women. In residents aged ≥ 50 years in the same population, Nambunmee et al. [10] reported that FECa, which increased with increased urinary Cd, was closely associated with an increase in urinary NTx, particularly in women. In the female participants in the current study, the prevalence of bone metabolic disorders based on a urinary NTx level ≥ 89.0 (nM BCE/mM Crea) was high, more than 25%, but only FECa was significantly associated with the prevalence of bone metabolic disorders. This suggest that indirect effects of Cd exposure on Ca alterations associated with urinary Cd or blood Cd may be important mechanisms behind the effects of Cd on bone resorption in women.

Some studies have investigated the effects of low-level Cd exposure on bone resorption and PTH in women in Europe [19,20]. Schutter et al. [20] suggested that the direct bone toxicity of Cd exposure released Ca from bone, and this combined with decreased PTH to normalize serum Ca levels in women living in a Cd-polluted area in Belgium. In the current study, the first to investigate whole PTH in bone metabolism in the Thai population, we found that the whole PTH level inversely correlated with increased urinary Cd (Spearman ρ = 0.387, *p* < 0.01) only in women, as was seen in women in Belgium. These results suggest that PTH is lower in women with high Ca excretion but without severe renal tubular dysfunction, as indicated by increased urinary low molecular protein. In female residents living in Cd-polluted areas in Japan, decreased serum 1α,25-dihydroxyvitamin D and increased PTH was associated with renal tubular dysfunction in terms of both gender and its association with BMD and PTH [3], suggesting that alterations in vitamin D metabolism and PTH may be some of the effects on bone induced by Cd. However, it is possible that alterations in vitamin D metabolism and PTH might only be found among Japanese people who have a high mean urinary β2-MG level (7030 μg/g Crea in men and 9954 μg/g Crea in women), and not in Thai people in the current study (1658 μg/g Crea in men and 352 μg/g Crea in women).

Metabolic forms of vitamin D were not measured in the current study because of the lack of adequate sample volume. Urinary VitDBP was measured because increased excretion of VitDBP was reported in residents with renal tubular dysfunction due to Cd exposure in Japan [21]. Increased blood Cd was slightly but significantly associated with increased serum OC in men only in the current study, suggesting recent Cd exposure may increase bone formation as well as bone resorption. Only in women, increased urinary VitDBP was significantly associated with serum OC but association was not significant after adjusting serum Crea, indicating urinary VitDBP associated with increased urinary excretion by renal dysfunction. In Mae Sot residents, renal dysfunction, indicated by increased serum Crea, associated with Cd exposure was reported [9]. It was suggested that cadmium-induced renal dysfunction may alter the formation of the active form of vitamin D, 1,25-dihydroxy vitamin D, which occurs in the kidney. Further studies are necessary to determine whether increased serum OC results in osteomalacia and abnormal bone formation, because the range for increased OC observed in participants was narrow and because we had a limited number (5%) of male patients with itai-itai disease [22] and exposed male factory workers with osteomalacia [23,24].

## 5. Conclusions

Cd exposure, particularly blood Cd, influenced bone remodeling (both bone resorption and bone formation) in this population of older Thai men. In women, urinary Cd was associated with the bone resorption marker, but FECa was the factor most closely associated with the bone resorption marker, suggesting an indirect effect of Cd exposure on bone remodeling by altering Ca excretion into the urine.

## Figures and Tables

**Table 1:** Comparisons of factors related to bone metabolism and Cd exposure markers by gender.

Factors		Men (N = 61)		Women (N = 69)			Reference
		Mean	SD	Mean	SD	*p*-Val	Range
Age	years	71.7	4.2	70.6	3.8	0.109	
Height	cm	158.7	6.0	148.8	4.4	0.000	
Weight	kg	49.3	8.0	45.2	9.6	0.011	
BMI		19.6	2.9	20.5	4.0	0.141	
Urinary NTx	nM BCE/mM Crea	41.9	1.9	67.5	1.8	0.000	<54.3
	≥66.2 ^a^ (%)	11	(34.4)				
	≥89.0 ^b^ (%)			19	(27.5)		
Serum OC	ng/mL	6.7	3.4	7.7	4.6	0.164	<12.7
	≥12.7 ^c^ (%)	5	(8.2)	6	(8.7)	0.919	
whole PTH	pg/mL	14.4	5.8	16.1	6.9	0.144	8.3–38.7
Serum Crea	mg/dL	1.4	0.5	1.1	0.4	0.000	0.4–1.5
Urinary Crea	g/L	1.3	0.7	1.1	0.5	0.056	1–1.5
Urinary VitDBP	mg/g Crea	125.8	3.9	135.7	3.3	0.743	300–600
FECa	%	0.8	2.5	0.7	2.7	0.328	2–4
FEP	%	11.6	2.0	7.9	2.1	0.004	10–20
Blood Cd	g/L	9.4	5.7	5.8	3.0	0.000	<5
	≥10.0 (%)	26	(42.6)	5	(7.2)	0.001	
Urinary Cd	μg/g Crea	6.7	1.8	6.5	1.8	0.798	<5
	≥10.0 (%)	18	(29.5)	17	(24.6)	0.532	

Note: SD: standard deviation; *p*-val: *p*-value; BMI: body mass index; NTx: N-terminal telopeptide; OC: Osteocalcin; PTH: parathyroid hormone; Crea: creatinine; VitDBP: Vitamin D binding protein; FECa: Fractional excretion of calcium; FEP: Fractional excretion of phosphorus; Cd: cadmium; ^a^ lower limit value for men at a risk of bone metabolic disorders [16]; ^b^ lower limit value for postmenopausal women at a risk of bone metabolic disorders [16]; ^c^ upper range of healthy persons.

**Table 2:** Simple correlation analysis (Spearman’s rho) between bone markers and renal and exposure markers.

Sex	Renal/Exposure Markers	Urinary NTx		Serum OC	
		Corr Co.	*p*-Val	Corr Co.	*p*-Val
Men	PTH	0.222	0.094	0.286	0.029
	Serum Crea	−0.072	0.581	0.278	0.030
	Urinary VitDBP	0.212	0.111	0.214	0.106
	FECa	0.375	0.004	0.054	0.686
	FEP	0.076	0.572	0.151	0.290
	Urinary Cd	0.196	0.139	0.027	0.839
	Blood Cd	0.241	0.068	0.052	0.699
Women	PTH	−0.272	0.028	0.138	0.275
	Serum Crea	−0.362	0.002	0.320	0.007
	Urinary VitDBP	0.313	0.011	0.265	0.021
	FECa	0.614	0.000	0.114	0.365
	FEP	0.041	0.746	0.001	0.997
	Urinary Cd	0.407	0.001	0.120	0.101
	Blood Cd	0.273	0.028	0.205	0.101

Note: NTx: N-terminal telopeptide; OC: Osteocalcin; Corr Co.: Correlation coefficient; *p*-val: *p*-value; PTH: parathyroid hormone; Crea: creatinine; VitDBP: Vitamin D binding protein; FECa: Fractional excretion of calcium; FEP: Fractional excretion of phosphorus; Cd: cadmium.

**Table 3:** Multiple regression analysis of urinary NTx with relevant factors including Cd exposure markers.

Sex		Men				Women			
Model	Factors	β	75%CI		*p*-Val	β	75%CI		*p*-Val
			Lower	Upper			Lower	Upper	
Model 1	Age	−0.107	−0.354	0.139	0.385	0.015	−0.201	0.232	0.887
	Smoking habit	0.323	0.094	0.553	0.007	0.040	−0.152	0.232	0.676
	Drinking habit	0.048	−0.182	0.276	0.683	−0.105	−0.297	0.086	0.275
	BMI	−0.186	−0.406	0.034	0.096	−0.145	−0.360	0.070	0.181
	PTH	0.246	0.003	0.489	0.047	−0.002	−0.205	0.202	0.985
	Serum Crea	0.001	−0.293	0.294	0.996	−0.091	−0.313	0.131	0.414
	FECa	0.254	0.01	0.498	0.041	0.420	0.194	0.647	0.000
	Urinary VitDBP	0.195	−0.078	0.468	0.157	0.138	−0.086	0.361	0.222
	Urinary Cd	0.373	0.152	0.595	0.001	0.243	0.030	0.456	0.026
Model 2	Age	−0.054	−0.295	0.188	0.658	−0.003	−0.231	0.225	0.976
	Smoking habit	0.324	0.099	0.548	0.006	0.030	−0.174	0.234	0.767
	Drinking habit	0.023	−0.200	0.246	0.836	−0.097	−0.298	0.104	0.340
	BMI	−0.118	−0.333	0.097	0.276	−0.207	−0.422	0.009	0.060
	PTH	0.270	0.030	0.509	0.028	−0.040	−0.248	0.169	0.704
	Serum Crea	−0.112	−0.392	0.167	0.424	−0.100	−0.330	0.131	0.390
	FECa	0.310	0.067	0.553	0.013	0.450	0.213	0.687	0.000
	Urinary VitDBP	0.265	−0.001	0.531	0.051	0.171	−0.059	0.402	0.142
	Blood Cd	0.408	0.190	0.627	0.000	0.068	−0.144	0.279	0.525

Note 1 : β: standardized beta; CI: confident interval; smoking habit: present smoker; drinking habit: regularly drinking; BMI: body mass index; NTx: N-terminal telopeptide; PTH: parathyroid hormone; Crea: creatinine; VitDBP: Vitamin D binding protein; FECa: Fractional excretion of calcium; Cd: cadmium; Note 2: adjusted r^2^ = 0.354 (*p* < 0.001) for Model 1 in men, adjusted r^2^ = 0.442 (*p* < 0.001) for Model 1 in women adjusted r^2^ = 0.381 (*p* < 0.001) for Model 2 in men, adjusted r^2^ = 0.396 (*p* < 0.001) for Model 2 in women.

**Table 4:** Multiple regression analysis of serum osteocalcin with relevant factors including Cd exposure markers.

Sex		Men				Women			
Model	Facotrs	β	75% CI		*p*-Val	β	75% CI		*p*-Val
			Lower	Upper			Lower	Upper	
Model 1	Age	0.190	−0.092	0.472	0.183	0.059	−0.182	0.301	0.626
	Smoking habit	0.171	−0.092	0.434	0.198	−0.049	−0.264	0.165	0.647
	Drinking habit	0.047	−0.215	0.309	0.719	−0.053	−0.267	0.161	0.623
	BMI	0.150	−0.102	0.403	0.237	−0.308	−0.548	−0.068	0.013
	PTH	0.077	−0.202	0.355	0.583	0.086	−0.141	0.313	0.452
	Serum Crea	0.311	−0.025	0.648	0.069	0.388	0.140	0.635	0.003
	FECa	0.021	−0.258	0.301	0.880	0.183	−0.070	0.436	0.153
	Urinary VitDBP	0.152	−0.161	0.465	0.333	0.176	−0.074	0.426	0.164
	Urinary Cd	0.200	−0.054	0.454	0.120	0.050	−0.188	0.287	0.678
Model 2	Age	0.223	−0.053	0.500	0.111	0.061	−0.184	0.307	0.619
	Smoking habit	0.170	−0.087	0.427	0.191	−0.045	−0.264	0.174	0.683
	Drinking habit	0.037	−0.218	0.293	0.771	−0.055	−0.271	0.161	0.612
	BMI	0.191	−0.056	0.437	0.127	−0.324	−0.556	−0.092	0.007
	PTH	0.101	−0.174	0.375	0.465	0.076	−0.148	0.301	0.498
	Serum Crea	0.250	−0.070	0.571	0.123	0.388	0.140	0.636	0.003
	FECa	0.063	−0.215	0.341	0.651	0.197	−0.058	0.452	0.128
	Urinary VitDBP	0.193	−0.112	0.498	0.210	0.184	−0.064	0.432	0.142
	Blood Cd	0.273	0.023	0.523	0.033	−0.019	−0.246	0.209	0.869

Note 1 : β: standardized beta; CI: confident interval; *p*-val: *p*-value; smoking habit: present smoker; drinking habit: regularly drinking; BMI: body mass index; OC: Osteocalcin; PTH: parathyroid hormone; Crea: creatinine; VitDBP: Vitamin D binding protein; FECa: Fractional excretion of calcium; Cd: cadmium; Note 2: adjusted r^2^ = 0.151 (*p* < 0.039) for Model 1 in men, adjusted r^2^ = 0.303 (*p* < 0.001) for Model 1 in women adjusted r^2^ = 0.186 (*p* < 0.05) for Model 2 in men, adjusted r^2^ = 0.301 (*p* < 0.001) for Model 2 in women.

**Table 5:** Risk factors for bone metabolic disorders, indicated by urinary NTx, analyzed using logistic regression model.

Model	Factors	OR	75% CI		*p*-Val
			Lower	Upper	
**Men** (total N = 61)	Urinary NTx ≥ 66.2 ^a^	(N = 11)			
Model 1	PTH	0.9	0.8	1.2	0.648
	Serum Crea	2.2	0.1	36.0	0.591
	FECa	37.9	1.5	948.9	0.027
	Urinary VitDBP	4.4	0.6	30.0	0.134
	Urinary Cd < 10	1.0			
	≥10 μg/g Crea	10.1	1.1	90.8	0.039
Model 2	PTH	1.0	0.8	1.2	0.989
	Serum Crea	2.1	0.1	44.3	0.633
	FECa	67.3	2.0	2259	0.019
	Urinary VitDBP	4.6	0.6	37.0	0.156
	Blood Cd < 10	1.0			
	≥ 10 μg/L	23.2	1.5	354.7	0.024
**Women** (total N = 69)	Urinary NTx ≥ 89.0 ^b^	(N = 19)			
Model 1	PTH	0.9	0.7	1.0	0.122
	Serum Crea	0.1	0.0	2.8	0.191
	FECa	5.8	0.7	48.3	0.106
	Urinary VitDBP	2.2	0.5	9.7	0.305
	Urinary Cd < 10	1.0			
	≥10 μg/g Crea	0.9	0.1	6.3	0.856
Model 2	PTH	0.9	0.7	1.0	0.107
	Serum Crea	0.1	0.0	2.6	0.171
	FECa	5.8	0.8	41.9	0.083
	Urinary VitDBP	2.4	0.5	10.7	0.263
	Blood Cd < 10	1.0			
	≥10 μg/L	0.5	0.0	10.7	0.633

Note 1 : N: number of subjects; NTx: N-terminal telopeptide (nM BCE/mM Crea); OR: Odds ratio; CI: confident interval; *p*-val: *p*-value; Covariates including age; smoking habit; drinking habit; and BMI: body mass index; PTH: parathyroid hormone; Crea: creatinine; VitDBP: Vitamin D binding protein; FECa: Fractional excretion of calcium; Cd: cadmium; Urinary Cd (μg/g Crea); Blood Cd (μg/L); Note 2: ^a^ lower limit value for men at a risk for bone metabolic disorders [16]; ^b^ lower limit value for postmenopausal women at a risk for bone metabolic disorders [16].
